# Supplementary material for: Therapeutic Efficacy of YM155 to Regulate an Epigenetic Enzyme in Major Subtypes of RCC
Source: Int J Mol Sci. 2023 Dec 22;25(1):216. doi: 10.3390/ijms25010216 (PMC10779260; doi:10.3390/ijms25010216)
Supplement: Supplementary file 1 [file ijms-25-00216-s001.zip › ijms-2709132-supplementary.pdf]

Supplementary data

Supplementary Table S1. List of the primer sequences used for RT-PCR studies.

	Sequence (5'→3')
RT-GAPDH-f	GAG TCA ACG GAT TTG GTC GT
RT-GAPDH-r	TGG AAG ATG GTG ATG GGA TT
RT-BIRC5-f	GAC GAC CCC ATA GAG GAA CAT A
RT-BIRC5-r	TTT CCT TTG CAA TTT TGT TCT TG
RT-EP300-f	CGC TTT GTC TAC ACC TGC AA
RT-EP300-r	TGC TGG TTG TTG CTC TCA TC
RT-EZH2-f	GAC CTC TGT CTT ACT TGT GGA GC
RT-EZH2-r	CGT CAG ATG GTG CCA GCA ATA G
ChIP-BIRC5 promoter-f	CCA CCG CAT CTC TAC ATT CA
ChIP-BIRC5 promoter-r	TTT CCT TTG CAA TTT TGT TCT TG

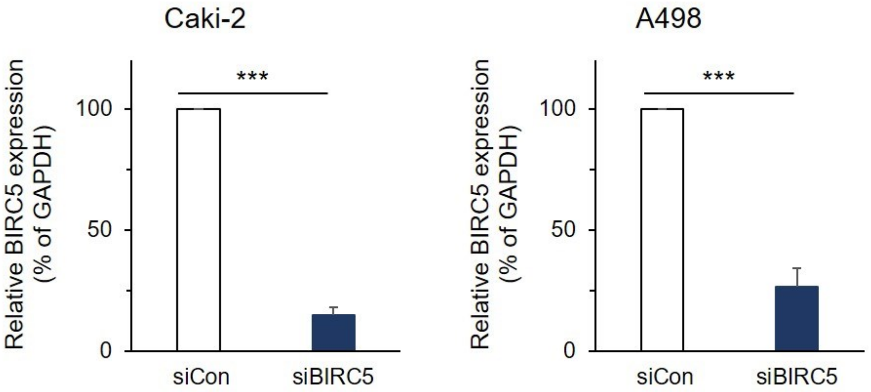

Supplementary Figure S1. BIRC5 mRNA levels were assessed by qRT-PCR in a second ccRCC cell line, Caki-2, and a second pRCC cell line, A498, transfected with the indicated siRNAs. Results represent mean ± SD of three independent experiments (***p<0.001 compared to siCon, t-test).

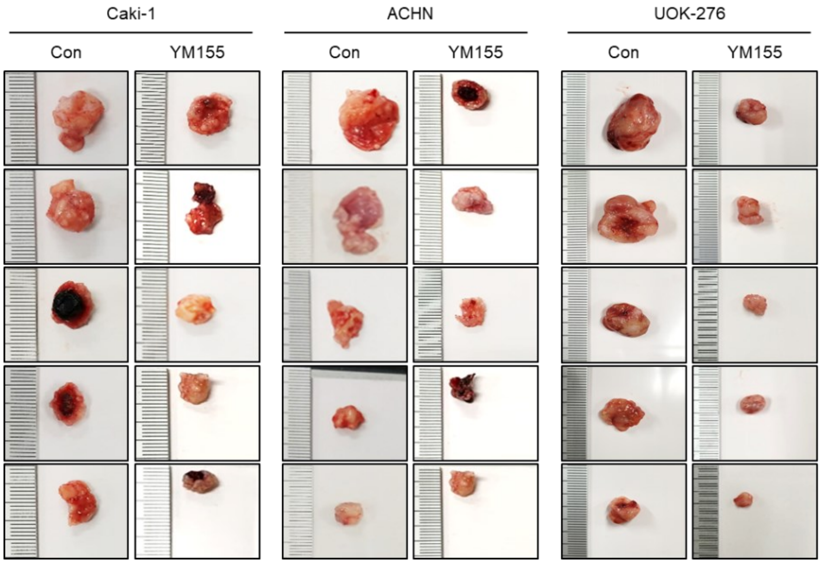

Supplementary Figure S2. Representative images of xenografts in the control and YM155 treated groups injected with Caki-1, ACHN, and UOK-276 cells at the end of the experiment.
